# Enhancing Quality of Resident Care and Staff Efficiency Through Implementation of Sensors in the Long-Term Care Setting: A Multi-Site Mixed-Methods Study

**DOI:** 10.3390/s25216795

**Published:** 2025-11-06

**Authors:** Shannon Freeman, Santiago Otalvaro Zapata, Matthew J. Sargent

**Affiliations:** 1 School of Nursing, University of Northern British Columbia, Prince George, BC V2N 4Z9, Canada; otalvaro@unbc.ca; 2 Centre for Technology Adoption for Aging in the North, University of Northern British Columbia, Prince George, BC V2N 4Z9, Canada; matt.sargent@unbc.ca

**Keywords:** long-term care, sleep disturbances, older adults, nursing, gerontology, sleep, sensors, evaluation

## Abstract

Introduction: Individuals residing in long-term care facilities (LTCFs) often experience poor sleep quality. Emerging sensor technologies may improve resident sleep quality and reduce staff workload. This evaluation assessed the impact of a bed sensor technology on LTCF staff experiences and resident outcomes. Methods: A mixed-methods evaluation examined the impact of a pilot implementation of Toch Sleepsense, a non-wearable sensor placed under residents’ beds, which monitors sleep patterns, movement, and vital signs. Data were gathered from staff surveys, interviews, and focus groups from three LTCFs in Western Canada. Descriptive statistics of survey data and thematic analysis of qualitative survey responses and focus groups were used to identify themes in staff experiences with Toch Sleepsense. Results: Staff valued the utility of Toch Sleepsense in providing alerts that support timely interventions and fall prevention. Staff further recognized the value of sensor devices in decreasing repetitive nighttime checks and providing vital sign monitoring. Toch Sleepsense data informed care planning and improved resident comfort. Inconsistent internet connectivity, sensor realignments, and limited training posed challenges to reliability. Conclusions: Sensor technologies like Toch Sleepsense show potential to improve safety, support staff workload management, and improve care practices. Sustained benefits require reliable technical infrastructure, comprehensive staff training, and strong leadership support.

## 1. Introduction

The global population of older adults is rapidly increasing, posing significant challenges for healthcare, social services, and economic systems worldwide. The World Health Organization predicts that the proportion of individuals aged 60 and older will increase from 12% in 2015 to approximately 22% by 2050, bringing the total population of adults aged 60 and older to over 2 billion [1]. Chronic diseases are leading causes of morbidity and mortality for older adults, intensifying the demand for specialized long-term care (LTC) services [2]. Consequently, many older adults with complex health conditions, functional impairments, and cognitive decline increasingly rely on institutional care settings to meet their daily needs [3].

Canada mirrors these global trends. Approximately 25% of Canadians are projected to be aged 65 or older by 2036, which will significantly impact healthcare infrastructure and service planning [4]. This demographic shift will lead to higher long-term care facility (LTCF) populations as older adults’ healthcare needs surpass the support capacities of informal or community-based care arrangements [5]. However, this transition into LTCF settings often introduces challenges to residents’ well-being, including disrupted sleep patterns and exposure to stressors such as nighttime noise and artificial lighting [6]. These factors negatively influence sleep quality, impacting resident’s overall health, cognitive function, and psychological well-being [7,8,9]. Poor sleep further exacerbates risks of falls, daytime impairment, agitation, and accelerated cognitive decline, placing considerable strain on caregivers and facility operations [10].

Given these critical issues, improving sleep quality and resident safety within LTCFs has become an urgent priority. Effectively addressing sleep disturbances and falls in LTCFs is essential for enhancing resident safety and well-being as well as for optimizing facility management and reducing caregiver burden. Emerging technological innovations offer potential solutions to these long-standing issues in LTCFs.

There has been much research examining the use of wearable and non-wearable sensors deployed within a range of home environments from supporting older adults to age independently in place in the community to supporting staff to provide high quality and efficient care in health care environments including LTCFs and acute care settings [11]. A scoping review of 30 studies examining sleep quality using non-wearable sleep trackers found that assessments may facilitate provision of quality care in both home-based and clinical environments [11]. Smart sensing devices are often combined with predictive modeling techniques (e.g., machine learning algorithms) to assess activities and routines.

There are advantages and challenges associated with use of wearable and non-wearable technologies. For example, Narasimhan et al. note that wearable sensors have higher localization accuracy and tracking yet are more intrusive in contrast to non-wearable sensors that are unobtrusive and useful in continuous monitoring over time [12]. For older adults, and individuals with cognitive impairment, use of wearable sensors can be challenging as they require users to agree to and remember to wear the device and in many cases require the user to charge the device or replace batteries [13,14]. In contrast, non-wearable devices are often considered less intrusive as they can capture data on the individual as they exist in their home environment without requiring adaptation or change to daily routines [12].

Objective sleep monitoring of older adults in their home sleeping environment is important but can be challenging as the use of polysomnography, the gold-standard for sleep disorder diagnosis, can be limited [15,16]. In a scoping review examining sleep quality assessment, Yamakawa et al. describe the breadth of existing sleep monitoring review studies which examine wearable and non-wearable sleep technologies and note that reviews on the subject have been published from 2015 to 2023 [11,17,18,19,20,21]. However, Yamakawa et al. also conclude that the sleep parameters looking into the quality of sleep vary across the 30 studies that they examined.

**Figure 1:**
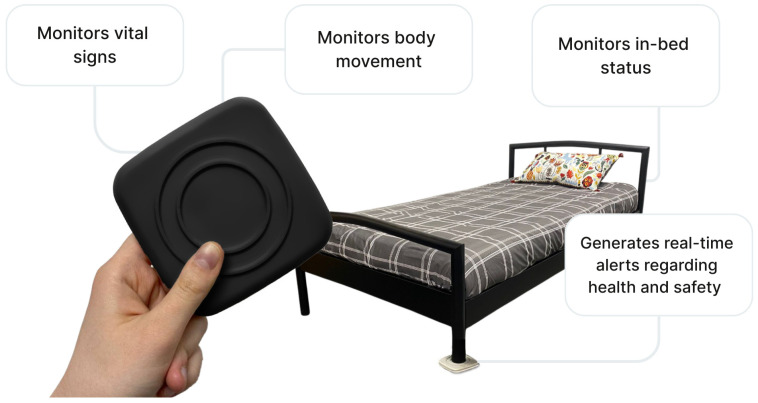
Technical Features of Toch Sleepsense.

Technologies to monitor sleep, including sensor-based monitoring systems, have been identified as valuable tools for enhancing resident safety, reducing falls, and promoting better sleep through continuous, non-invasive monitoring [22,23]. Non-wearable sensors have been shown to be effective from a clinical practice perspective as they can detect activity patterns and determine what is normal and abnormal over time [12]. Research has extensively studied sleep quality and sleep disorders (e.g., sleep apnea) using sensor devices [24]. Research has shown that bed sensors can be used to capture multiple measurements, including regular and irregular sleep patterns and frequency of bed exits, which can inform the health and well-being of LTCF residents, in particular for residents with dementia [13]. Schütz et al. found that monitoring of body movements in bed by contactless bed sensor devices over time can allow for proactive screening and monitoring of health and can serve as an easy to acquire digital biomarker [24]. One example is Toch Sleepsense, a sensor system designed for placement under residents’ beds, which integrates with existing nurse call and smart care systems [25]. This technology, shown in Figure 1, enables staff to conduct virtual bed checks by providing information about residents’ vital signs and whether residents are in or out of bed, potentially reducing nighttime disruptions for residents and decreasing caregiver workload. By facilitating remote monitoring and generating real-time alerts, it has the potential to contribute to improved resident safety, sleep quality, and overall care management.

Understanding practical solutions and interventions that improve resident care and sleep management in LTCFs is vital, particularly as resident needs grow more complex and staffing pressures intensify. While previous evaluations have examined the perceived utility and early implementation potential of Toch Sleepsense technology, primarily in urban settings [23,26], limitations remain in understanding of how such technology is implemented and how it performs in routine practice, particularly within the realities of everyday care of LTCF settings. This study addresses this gap by examining the implementation of Toch Sleepsense in three LTCFs. By focusing on frontline staff perspectives after real-world integration, this study captures the lived experience of staff using sleep sensor technology in everyday care. The findings provide practical insight into the usability, effectiveness, and sustainability of this innovation in residential care environments, where staffing models, infrastructure, and resident needs differ significantly from hospital-based settings.

## 2. Materials and Methods

This research employed a convergent mixed-methods design to evaluate the implementation and perceived impact of Toch Sleepsense in three LTCFs in Western Canada. This approach was chosen to concurrently collect qualitative and quantitative data, allowing for data triangulation to promote a nuanced understanding of the complex implementation processes of the technology in LTCF settings.

**Figure 2:**
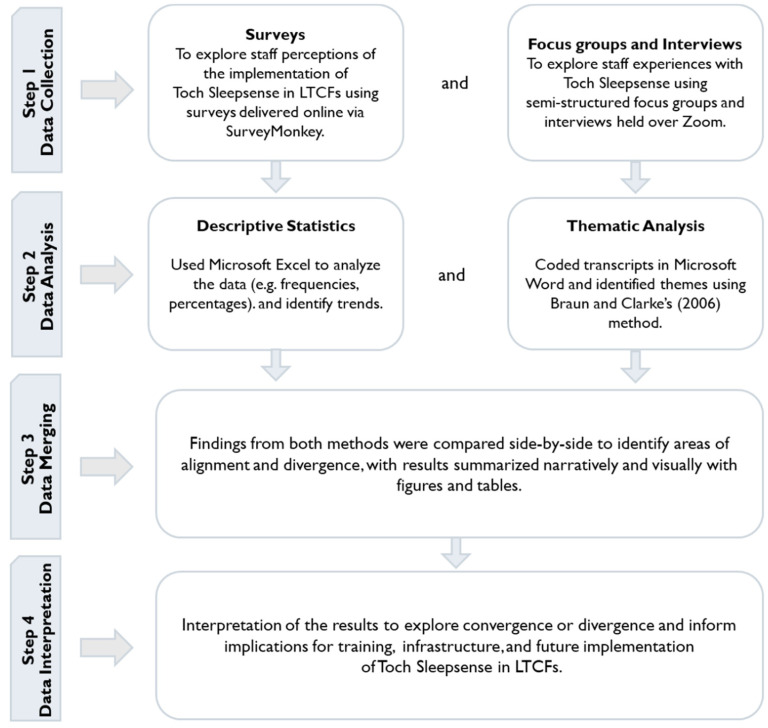
Convergent Mixed-Methods Design for Toch Sleepsense [28].

Guided by Creswell and Plano Clark’s mixed-methods framework [27], this study’s qualitative and quantitative components were integrated through a structured, multi-stage process shown in Figure 2. Both data types were gathered in parallel to ensure temporal alignment, and subsequent analysis was conducted independently for each stream of data. Once analyzed, findings were consolidated to facilitate cross-method comparison, enabling the identification of consistent patterns, contradictory insights, and complementary perspectives. This comparative synthesis informed the final interpretive stage, in which the combined results were examined to derive a comprehensive understanding of the technology’s impact across settings.

Participants included frontline caregiving staff and facility management from the three LTCFs in which Toch Sleepsense was implemented. Eligibility criteria included being employed at one of the participating LTCFs in which Toch Sleepsense had been trialed and having direct experience using Toch Sleepsense since its implementation. Staff must have been able to complete an online survey delivered in English or participate in Zoom-based focus groups or interviews in English to participate.

The Toch Sleepsense sensors were obtained from TochTech Technologies, a biotechnology company based out of Vancouver, Canada. Installation and calibration of Toch Sleepsense sensors, as well as network integration, were managed by the technology developer Toch Technologies (https://www.tochtech.com/ (accessed on 29 July 2025)) and were completed within two days at each site. Staff training with Toch Sleepsense occurred during installation, though training attendance varied due to staff availability constraints. For two of the LTCF sites (Site A and Site B), Toch Sleepsense was implemented for a one-month trial period in the spring of 2024. At Site A, 8 Toch Sleepsense devices were implemented and have remained in use at the facility since. At Site B, 21 Toch Sleepsense devices were implemented and then subsequently removed from the facility following the end of the trial period. At Site C, 139 Toch Sleepsense devices were implemented in the facility in spring 2022 and the devices have remained in use at the LTCF since.

Participants were recruited from three LTCFs where the Toch Sleepsense system had been implemented. Eligible participants included nursing staff, care aides, and managers who had experience using the Toch Sleepsense system as part of routine care. Recruitment began in summer 2024 for Site A and Site B and in winter 2024 for Site C. For the surveys, facility directors distributed email invitations to all eligible staff, containing a link to an online survey hosted on SurveyMonkey. For the focus groups and interviews, facility directors disseminated invitations, encouraging interested individuals to contact the research team directly to participate.

For the quantitative research component, an online survey was administered to eligible prospective participants through SurveyMonkey (See Text S1). Survey items included statements related to workload impact, usability, and overall satisfaction with the technology (e.g., “I find the Toch Sleepsense dashboard easy to understand”, “Toch Sleepsense technology helps keep residents safe”), which participants indicated their level of agreement with on five-point Likert scales. In addition, the survey contained open-ended questions which allowed participants to provide more fulsome details about their experiences with Toch Sleepsense. This survey is available in the Supplementary Materials (See Text S1). Four semi-structured focus groups were conducted via Zoom. One focus group was held with frontline LTCF staff at Site C, and three focus groups were held with LTCF management, with one each at Site A, Site B, and Site C. Each focus group explored participants’ experiences with Toch Sleepsense, including their perceptions of its effect on staff workload, and its perceived influence on resident care. Focus groups were facilitated by trained members of the research team, lasted approximately 60–90 min, and were audio-recorded and transcribed verbatim. Participants also completed a short demographic questionnaire before their session. One interview was held with a manager from Site C who was unable to attend the Site C focus group but wished to participate. This interview followed the same semi-structured script used in the focus group (See Text S2), and the interview was conducted by a trained member of the research team, audio recorded, and transcribed verbatim. Semi-structured focus group scripts are available in the Supplementary Materials (See Text S2).

Consistent with Creswell and Plano Clark’s [27] guidelines for convergent mixed-methods research, quantitative and qualitative findings were integrated during interpretation and presentation of results. Points of convergence and divergence between data sets were identified, critically assessed, and discussed between the manuscript authors, allowing for a comprehensive understanding of Toch Sleepsense’s real-world impact and implementation complexities within LTCF settings.

Quantitative survey responses were analyzed using Microsoft Excel to prepare descriptive statistics. Frequency distributions and percentages were calculated to identify key patterns in responses to Likert-scale statements. Qualitative focus group transcripts were thematically analyzed following Braun and Clarke’s methodology [28]. The analysis involved systematically reviewing transcripts to identify notable patterns and develop themes that captured key insights related to the perceived benefits, challenges, and contextual factors influencing the adoption of Toch Sleepsense.

To enhance trustworthiness of this work, careful integration of qualitative and quantitative data sources were undertaken including review by all research team members. Further, transcripts were independently coded and reviewed by the second and third author and consensus on interpretation was obtained to ensure credibility and reliability in alignment with Creswell and Plano Clark’s guidelines [27]. To reduce the risk of confirmation bias, research team debriefing sessions were held regularly throughout data analysis, integration, and interpretation to allow for discussion and reflection.

This study was reviewed by the University of Northern British Columbia Research Ethics Board (Reference: 6009749), the University of British Columbia’s harmonized RISe platform (Reference: H23-04033), and by the National Research Council’s Research Ethics Board (National Research Council Industrial Research Assistance Program Project: 983747). Survey participants provided informed consent electronically before beginning the survey. Focus group and interview participants provided written informed consent forms and verbal consent was reaffirmed prior to study participation.

## 3. Results

### 3.1. Sociodemographic Characteristics of Participantstabk

An overview of the sociodemographic characteristics of study participants is shown in Table 1. Of note, most participants were between the ages of 35–54 for both online survey (64%) and the focus group (50%) participants. Most participants had more than 1 year of experience working in their current role (84%—online survey, 86%—focus group) and more than 1 year of experience working in the LTC sector (88%—online survey, 86%—focus group).

### 3.2. Implementation Outcomes and Thematic Findings

**Figure 3:**
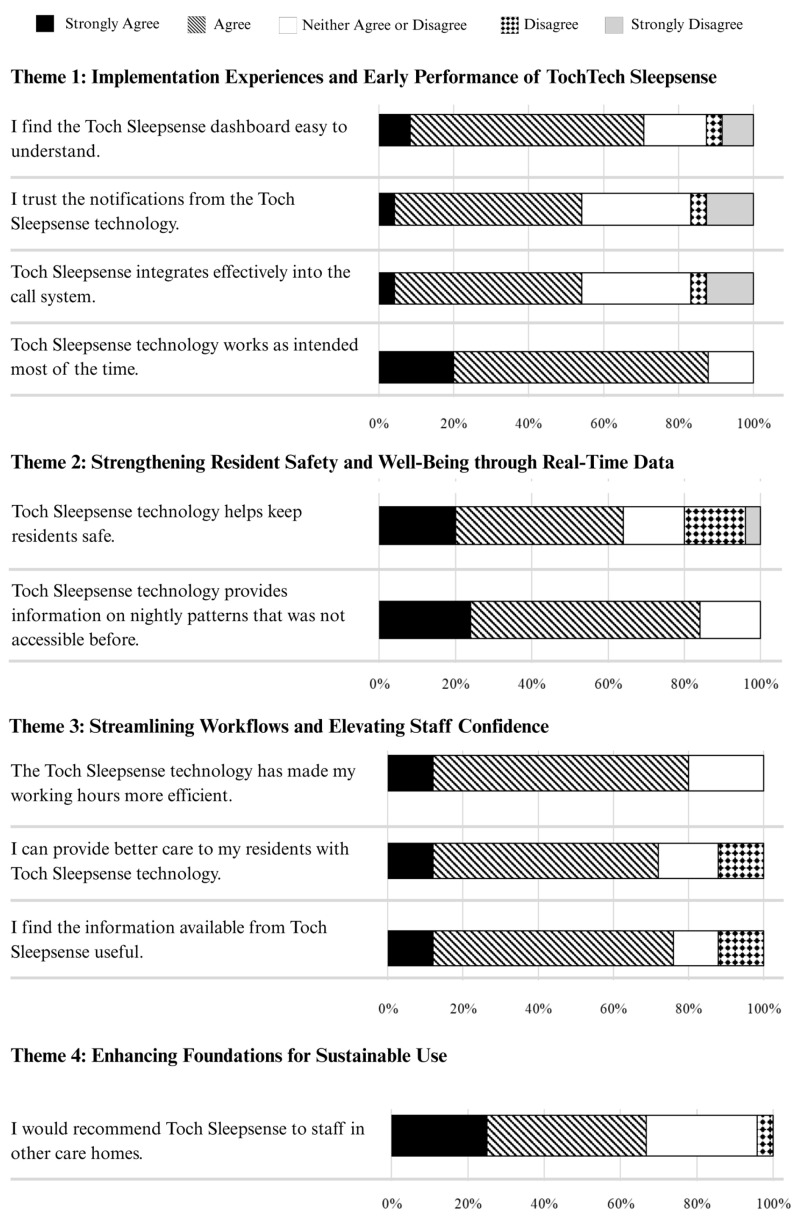
Convergence of Staff Survey Data and Qualitative Themes on Implementation (*N* = 25).

Staff perspectives on Toch Sleepsense were captured through both surveys and focus groups, with strong alignment emerging between quantitative responses and qualitative insights. As shown in Figure 3, most staff reported favorable experiences across areas such as usability, care quality, and workflow efficiency. These survey responses reflected and reinforced the qualitative findings, demonstrating convergence across data sources and highlighting key patterns in how the technology was perceived and integrated in day-to-day care.

#### 3.2.1. Theme 1: Implementation Experiences and Early Performance of Toch Sleepsense

Participants recognized the potential of Toch Sleepsense to enhance resident monitoring, yet the degree to which Toch Sleepsense was adopted varied substantially between the three sites. Factors that contributed to discrepancies in adoption included site-level training, infrastructure, and leadership supports. Survey data showed that across all sites, 88% of survey respondents responded either “Agree” or “Strongly Agree” to the statement “I find the Toch Sleepsense dashboard easy to understand”, and 68% responded either “Agree” or “Strongly Agree” to the statement “Toch Sleepsense technology works as intended most of the time”. These insights closely align with focus group accounts highlighting how accessible design can foster quick uptake, whereas connectivity gaps or insufficient training can obstruct performance and day-to-day use. In Site A, staff described a smooth implementation, reporting minimal technical frustration and a user-friendly interface. A focus group participant noted:

Really easy to use. New staff haven’t been coming to me 46 times asking how to use it, so that says a lot. Some people are not really great with technology, but they’re still able to bring it up. (Participant 1, Focus Group 1, Management).

In contrast, limited structured orientation and a lack of a knowledgeable champion to spearhead the implementation impeded the technology’s integration at Site B. Participants who had limited support throughout the implementation process failed to realize the full capabilities of Toch Sleepsense’s functions. The reliability of Toch Sleepsense also depended heavily on stable connectivity. Some staff described the need to unplug and reconnect the device after minor outages, and others reported issues such as the Toch Sleepsense devices consuming substantial Wi-Fi bandwidth, which resulted in delayed notifications. Temporary Wi-Fi disconnections and network overloads, caused by the sensors sending updates every minute, resulted in delayed alerts and missed data. As one manager at Site A explained, these limitations impacted the timeliness of staff notifications and ultimately affected trust in the system:

[The Wi-Fi] was overloaded because the sensor needs to send an update every minute… So, if you have really slow Wi-Fi and everyone is using the same Wi-Fi then you’re receiving a late response. That it would impact the alert that the staff are receiving. (Participant 2, Focus Group 1, Management).

Additionally, maintenance tasks and housekeeping activities further complicated sustained adoption. For instance, routine bed movement during housekeeping frequently dislodged sensors or disrupted their calibration, necessitating tedious recalibration processes. Despite these hurdles, participants in sites with more successful implementation processes appreciated the insights gained from Toch Sleepsense, including the real-time alerts and comprehensive dashboard analytics, which they felt enabled prompt responses to urgent situations.

Overall, mixed sentiments emerged in response to survey items assessing ease of use, trust in notifications, and overall reliability, where participants endorsed survey items such as “I find the information from Toch Sleepsense useful” (80% of respondents answered “Agree” or “Strongly Agree”). Responses to items related to dashboard integration and notification reliability varied, with 54% of participants responding “Agree” or “Strongly Agree” to the statement “I trust the notifications from the Toch Sleepsense technology”, and 54% responding “Agree” or “Strongly Agree” to the statement “Toch Sleepsense integrates effectively into the call system”. In sum, these convergent findings, spanning both numerical data and qualitative experiences, indicate that while user-centered design can speed initial acceptance, sustained success hinges on adequate connectivity, strategic education, and organizational buy-in.

#### 3.2.2. Theme 2: Strengthening Resident Safety and Well-Being Through Real-Time Data

Staff members described Toch Sleepsense as helpful for understanding and supporting residents’ nighttime needs, largely due to the insights it offered into individuals’ sleep patterns, heart rates, and respiratory activity. Eighty-four percent of survey participants responded either “Agree” or “Strongly Agree” to the statement “The Toch Sleepsense technology provides information on nightly patterns that was not accessible before”, while 64% of survey participants responded either “Agree” or “Strongly Agree” to the statement “Toch Sleepsense technology helps keep residents safe”. These numbers echoed focus group anecdotes from frontline staff, who appreciated receiving early notifications of whether a resident, particularly those at high risk for falls, was out of bed. One registered nurse in a focus group recalled the following:

I think of a gentleman that used to live here who used to have falls and sometimes before [Toch Sleepsense] was in would have to kind of slide across the floor to get to the door and call for help if he wasn’t remembering to push the call bell. (Participant 2, Focus Group 1, Registered Nurse).

Further, staff used the sleep data from the Toch Sleepsense dashboard to tailor activities after a restless or agitated night, or modify care plans when a resident showed signs of health risks such as sleep apnea. A manager at Site A described how Toch Sleepsense data were used to inform daytime activities:

For example, today nobody slept very well last night. So today they haven’t done any of their chair exercises. Instead, they watched a movie, had, you know, very relaxed type of day. And I think that if we were to not know that information and maybe push them to do activities that were a little bit much, that’s when you see behaviors. And yeah, so it sounds like that’s, you know, that’s an area where it’s really helps to kind of inform the care plans in the overall home. (Participant 1, Focus Group 1, Management).

Staff also highlighted how aggregated Toch Sleepsense data improved daily care plans, especially for medication reviews and personalized schedules. Rather than relying on anecdotal evidence, caregivers could pinpoint exactly when residents slept poorly, then collaborate with physicians to address potential side effects of medications or dosage adjustments. As one manager noted:

We can communicate it to [physicians] through objective data, like hey, there’s actually in the sleep score that says that very low sleep and they’re waking up every hour at night and yet you ordered risperidone 50 milligrams for sleep. It’s not enough. And it’s just that having to have that avenue for conversation with the data with us, as nurses, is really important because then the physicians themselves like “Oh she’s not talking nonsense” I was like, I’m just like, “No, this is not a subjective thing. It’s actually objective now.” (Participant 1, Focus Group 3, Management).

However, some participants noted that the system’s effectiveness depended on reliable connectivity and accurate sensor readings, particularly for residents with atypical weights or conditions. For instance, staff described a situation where Toch Sleepsense gave inaccurate information due to one resident being very light weight, and another situation where two residents shared a bed which caused Toch Sleepsense to deliver inaccurate information. Despite these conditions, most staff reported that early detection of risks and clearer data on sleep quality brought reassurance for both frontline staff and families seeking answers about a loved one’s nighttime care. A participant at Site C shared:

Because we talk to our families. They’re actually quite impressed with the technology in terms of knowing that their family member is being monitored but not interrupted from their sleep… when people have falls, especially if it’s at nighttime, family members would say to me “Well, how long were they on the floor?” … Now I can tell them right away. Now we get an alert and we’re responding right away. (Participant 1, Focus Group 3, Management).

#### 3.2.3. Theme 3: Streamlining Workflows and Elevating Staff Confidence

A prominent theme among participants was the sensor’s capacity to reduce repetitive tasks, which strengthened efficiency and confidence during shifts. Staff members highlighted how real-time notifications replaced frequent, intrusive room checks. This allowed staff to prioritize other activities during their shifts while ensuring resident safety through virtual nighttime checks, reducing idle time and enhancing overall workflow. A manager at Site A remarked: “You can just have the laptop open while folding laundry or cleaning the kitchen … you’re getting notifications in real time of this person got up … and you pay attention to what’s going on at that time.” (Participant 1, Focus Group 1, Management).

Focus group participants across multiple facilities noted how having immediate insight into residents’ movements or vital signs helped them more effectively allocate their time. This was described as a key benefit of the sensor, as it reduced unnecessary room checks during rounds. This prevented nurses from disturbing residents, particularly those sensitive to noise or light. A licensed practical nurse at Site C noted:

So I would have to do hourly checks on residents. You know, opening the door to listen or see if they’re breathing. But sometimes they’re really sensitive, so they would wake up or maybe I’d have to get a bit closer to them and like actually shake them to make sure they’re okay. So Sleepsense is helpful in the way it saves the nurses a lot of time to focus on other responsibilities they might have at that moment. (Participant 2, Focus Group 3, Licensed Practical Nurse).

Survey data reinforced these observations. Seventy-six percent of survey participants responded either “Agree” or “Strongly Agree” to the statement “The Toch Sleepsense technology has made my working hours more efficient”, while 72% of participants responded either “Agree” or “Strongly Agree” to the statement “I can provide better care to my residents with Toch Sleepsense technology”.

However, despite staff members’ overall positive perceptions of Toch Sleepsense’s ability to aid with efficiency and safety of care, staff at sites with unstable Wi-Fi or limited training expressed neutral or skeptical views about the technology’s usefulness in improving workflow. They noted that connectivity issues, uncertainty about sensor accuracy, and intermittent calibration problems could make sensors difficult to use effectively. As a result, staff emphasized that effective integration, supported by reliable infrastructure and ongoing training, was essential. When these conditions were met, the sensors saved time, boosted staff confidence, and enhanced job satisfaction, fostering a more person-centered and responsive care environment. One manager at Site A highlighted the importance of dedicated leadership in this process, explaining:

There has to be some dedicated champion for some time within the facility. And I don’t mean full-time or even a part-time job. I’m saying an individual has to be freed up to have so much time to kind of just work with the staff and do the change management. This isn’t kind of the same as saying, you know, “Here, play with your phone and see what you think of this app.” It’s really understanding how it works and why. The rationale is why are we using this. How can it benefit me? How can it benefit the resident? (Participant 1, Focus Group 2, Management).

**Figure 4:**
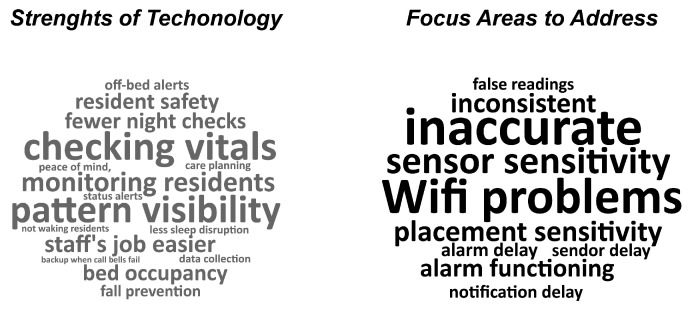
Word Clouds Depicting Staff-Identified Strengths and Improvement Areas for Toch Sleepsense.

Figure 4 shows word-cloud visualizations of staff feedback on the implementation of Toch Sleepsense. This word-cloud provides a visual depiction of common themes and ideas expressed by staff members when discussing Sleepsense. The size of the word in the figure represents the number of times this was mentioned by the participants. Participants consistently identified fall prevention (shown in large font under strengths of technology), followed by vital-sign monitoring, and the ability of the sensors to make their job easier as key strengths, as well as highlighting how real-time insights reduced intrusions into residents’ rest. At the same time, many expressed ongoing concerns about inconsistency (Shown in large font under focus areas to address), internet issues, and delayed alerts, which can hamper the technology’s potential if connectivity and training gaps remain unresolved. When the sensor functioned reliably and staff understood its features, they felt more assured, describing a setting where tasks were better prioritized, time-consuming hourly checks were reduced, and resident well-being remained paramount.

#### 3.2.4. Theme 4: Enhancing Foundations for Sustainable Use

Although most survey respondents expressed a willingness to recommend Toch Sleepsense to other care homes, participants also emphasized the need to address logistical and infrastructural barriers to realize the system’s full potential. A prominent issue was the necessity for allocating sufficient time and resources for a smooth implementation. Many staff members recounted feeling rushed during installation and training, which left them unable to fully grasp the system’s capabilities or troubleshoot issues in a timely manner. Participants stressed that leadership support, from a designated champion who could offer guidance and maintain momentum throughout the implementation process, was critical for sustaining interest, equipping new and current staff with the necessary skills, and coordinating ongoing system improvements.

Another recurrent theme was the requirement of robust infrastructure to avoid connectivity setbacks. While staff valued the convenience of real-time monitoring, the technology’s reliance on stable Wi-Fi magnified the impact of even minor network interruptions. Housekeeping staff and maintenance personnel needed clear instruction on sensor placement, which often proved challenging given diverse bed designs and daily bed movement. Participants noted that errors in calibration or alignment caused gaps in transmission of data from the sensors, undermining staff trust in the data. As a result, managers suggested scheduling dedicated sessions for non-clinical staff to reinforce awareness of how small misplacements or moving beds could lead to inconsistent readings.

## 4. Discussion

This study examined staff experiences with the implementation and adoption of Toch Sleepsense in three LTCFs in Western Canada. While the sensor technology was generally appreciated, and staff expressed clear interest in its continued use, its performance and perceived value were highly dependent on the structural conditions of each facility. Differences in training quality, leadership engagement, and infrastructure support shaped how effectively the system was adopted and integrated into routine care. Sites that had a dedicated champion and a structured implementation process described smoother transitions, greater interest from staff, and more reliable use of the system. In contrast, where implementation was rushed or training was inconsistent, staff reported confusion about the device’s purpose, difficulty troubleshooting issues, and frustration with unreliable connectivity.

Implementing new technologies in care settings is heavily influenced by organizational readiness, particularly the engagement of leadership and staff. In our study, the results revealed that the presence, or absence, of strong organizational support played a decisive role in how new sensor technology was received and utilized. When leadership actively supported implementation and clear roles were designated for oversight, the technology was more easily integrated into existing workflows and staff were more likely to explore the system’s full capabilities, troubleshoot minor issues, and rely on the data to inform care. In contrast, in settings where implementation lacked coordination or where staff had limited time and training, the technology often remained underused, and its value was not properly realized.

Numerous studies have also established that strong leadership and active participation among staff are essential for successful adoption, as they shape the culture of acceptance and problem-solving around new systems. For instance, van den Hoed et al. [29] emphasized that leadership engagement and internal alignment are critical facilitators of digital health adoption in long-term care homes. The study highlighted the importance of designating a champion who could coordinate staff engagement and serve as a reliable point of contact for troubleshooting, a strategy that was mirrored at sites with successful implementation in our present study. Similarly, Cruise et al. [30] noted that managers play a main role in advancing technology adoption in Canadian LTCFs, and managers prioritize ease of use, impact on workload, and perceived value when deciding whether to support the adoption of new technologies. These dimensions were echoed in our findings, where strong buy-in and interest were observed only when leaders and staff saw tangible benefits in terms of efficiency, safety, or care planning.

The importance of leadership and time allocation is further supported by Miake-Lye et al. [31], whose systematic review of implementation readiness frameworks identified inner setting characteristics, such as communication, leadership engagement, and implementation climate, as dominant factors influencing change adoption. These factors were evident in the present study, where staff frequently linked system misuse or frustration to a lack of training, time, or continuity. Facilities that introduced the new sensor technology in a rushed or unstructured way lacked the internal alignment needed for effective integration. This was especially evident in settings where non-clinical staff, such as housekeeping or maintenance, were excluded from training, leading to operational issues like dislodged sensors and unrecognized connectivity errors.

Staff described how the system enabled them to track residents’ heart and respiratory rates and in-bed status, leading to earlier identification of health concerns and tailored care planning. Importantly, staff used the sleep data to adjust daily activities and support medication reviews, allowing for a more person-centered approach. These findings highlight the value of continuous monitoring technologies in long-term care, offering clinical insights that might otherwise go unnoticed through routine observation alone.

This perspective aligns with a review from Yamakawa et al. [11], who reported that non-wearable sleep trackers offer reliable, real-time data on sleep quality and physiological trends in care settings. As in our study, their review emphasized the value of such tools for older adults who may resist wearable devices. However, Yamakawa et al. [11] also cautioned that inconsistencies in the sensor’s system may limit their clinical utility. Staff in our study echoed this concern, describing how misleading or delayed alerts or gaps in data occurred in cases where resident body weight was low or when bed movement interfered with sensor calibration.

Similarly, Kosse et al. [32] discussed how bed-based sensors may improve fall prevention through real-time alerts, but stressed that sensor technology alone cannot improve care outcomes without strong organizational structures, training, and workflow integration, a point reinforced by staff feedback in our study. In our study, while some care teams effectively used the sensor technology to streamline overnight checks and respond to high-risk events, others described disconnects between system potential and actual use due to limited training or unclear procedures around responding to alerts.

Our findings are consistent with previous literature, which found that the introduction of sensor-based alerts alone was not sufficient to significantly reduce falls in older hospital patients. A randomized controlled trial by Shorr et al. [33] found no significant difference in fall rates or related events between hospital units using bed alarm systems and those without. Likewise, Balaguera et al. [34] emphasized that the success of sensor-based care systems hinges not only on the technical accuracy of the device but also on its alignment with clinical workflows and the ability of staff to interpret and act on the data in real time. These studies highlight that without proper implementation strategies and staff engagement, the potential benefits of sensor technologies in fall prevention may not be fully realized.

Toch Sleepsense positively influenced staff workflow and perceived resident safety, particularly regarding night shift routines and resident safety monitoring. Staff reported that the technology reduced the need for intrusive physical checks during the night, enabling them to complete other tasks without compromising resident oversight. This perceived increase in efficiency, however, was closely tied to system reliability and organizational readiness. In settings where alerts were timely and training was sufficient, staff felt more confident and less anxious about missing critical events. Conversely, in environments with limited support or unreliable connectivity, staff described persistent concerns about whether the system could be trusted, adding to cognitive burden rather than alleviating it.

Our findings illustrate how sensor-based technologies can substantially alleviate nightly workload in LTCFs by reducing repetitive bed checks and enhancing data-driven care decisions. Wilfling et al. [35] observed that 78.4% of night-shift nurses in German nursing homes regularly face a significant burden from resident sleep disturbances, with few non-pharmacological strategies available to them. Our results build on that work by demonstrating how real-time alerts and aggregated reports from technologies like Toch Sleepsense can reduce guesswork around nighttime disruptions, allow staff to attend to more pressing tasks, and facilitate targeted interventions. However, such advantages do not automatically translate into lower stress or more efficient workflows if training, connectivity, and practical guidelines remain deficient. Wilfling et al. [36] found that although staff widely recognize the importance of nighttime care, fewer than a quarter of facilities have formal policy documents providing guidance on residents’ sleep at night, and over 90% of nurses reported never having received dedicated instruction in sleep management beyond their basic training. In our sample, Toch Sleepsense provided immediate insight and aided data-driven planning, yet its effectiveness hinged on staff understanding of the system’s alerts and the presence of coherent procedures to integrate sensor data into overall care routines.

In parallel, insights from another implementation study of Toch Sleepsense in a geriatric care setting reinforce how real-time occupant movement data can refine staff workflows and reduce unnecessary checks. Acosta et al. [23] showed that sensor alerts enabled frontline providers to reduce workload, respond proactively to changes in resident activity, and streamline communication among interdisciplinary teams. This aligns with our finding that immediate access to insights from the sensor data preserves staff time for higher priority tasks and instills confidence in the accuracy and timeliness of monitoring. Consequently, the combination of automatic alerts, aggregated sensor-based metrics, and proactive care strategies can alleviate some of the emotional and operational strain that traditionally accompanies resident supervision, especially during nightshifts, illustrating the broader potential for smart solutions to support both staff efficiency and resident well-being.

Ultimately, while sensor-based monitoring technologies like Toch Sleepsense can contribute meaningfully to resident safety and personalized care, their impact is mediated by contextual factors including reliability, workflow integration, and staff preparedness. The benefits observed in environments where implementation is well-supported include proactive risk management, reduced nighttime checks, and support for individualized care planning, demonstrating the value of these technologies when embedded appropriately into practice. However, the variability in outcomes across settings in this study underscores the importance of continued investment in training, infrastructure, and evaluation to ensure the technology supports routine care.

Study participants outlined several practical recommendations for maximizing the value of sensors technology use in the LTC setting and reducing the disruptions that can arise when such innovations are introduced. They consistently stressed the need to provide training beyond an initial orientation, suggesting periodic refresher sessions and active follow-up so staff remain adept at using and troubleshooting the system. Indeed, panels of LTC system leaders have suggested that ongoing, high-quality training is central to successful technology adoption in LTC settings [37]. In addition, participants highlighted the importance of dependable network connectivity, observing that frequent interruptions or slow data transmission can erode staff confidence in the system and its efficacy. These concerns align with broader evidence suggesting that dependable internet infrastructure is essential for sustaining staff engagement and ensuring the effective use of sensor-based monitoring technologies [38,39,40].

Another recommendation from our study concerns the appointment of a “technology champion” or point person to coordinate troubleshooting, collect staff feedback, and communicate with the technology’s support team. Relatedly, participants also emphasized the importance of including housekeeping and maintenance teams in any training or planning discussions. Frequent bed adjustments or rearrangements can easily lead to sensor misalignment, so having non-clinical staff informed about the system reduces accidental errors, in alignment with prior evidence on the benefits of interprofessional collaboration for implementation of technologies within LTCFs [41,42]. Finally, multiple staff emphasized the value of systematically reviewing sensor outputs, such as nighttime heart rate or respiratory trends, during clinical handovers or care plan meetings to prompt timely medication adjustments or changes in sleep routines. By following these suggestions and allowing them to inform the evidence base for organizational readiness, facilities can increase the likelihood that sensor-based monitoring will genuinely ease staff workloads and support more nuanced, person-centered care.

A key strength of this evaluation lies in its convergent mixed-methods approach, as staff perspectives were gathered through both an online survey and through focus groups. This inclusive design enabled triangulation of quantitative and qualitative data, capturing numerical indicators (e.g., Likert scale survey data related to perceived ease of use, trust in notifications) as well as rich, contextual insights about challenges like sensor dislodgement and connectivity lapses. Including staff from multiple LTCFs, each with varying degrees of technical infrastructure and organizational readiness, further enhances the study’s generalizability by showing how contextual factors can shape adoption outcomes. Additionally, the emphasis on firsthand accounts from direct-care staff and facility managers allowed for a multifaceted view of the technology’s real-world utility, illustrating how factors such as resident comfort and staff workflow intersect with technological performance.

Our study has several limitations. In the survey, participants from Site C were disproportionally represented, accounting for 76% of respondents. Due to the successful Toch Sleepsense implementation at Site C, our survey results portray opinions of Toch Sleepsense which may not reflect the consensus across all LTCFs in which the device has been trialed. Further, the short, one-month trial period in which the technology was implemented at Site B may not have fully captured longer-term changes in practice or potential improvements if staff were to have become more proficient with the technology. Though the mixed-methods design provides depth, the lack of direct resident-level outcomes (e.g., actual fall rates, measured sleep data) means that conclusions about clinical effectiveness must be inferred from staff-reported observations. While this study described staff perspectives and experiences using the technology, resident experiences as well as perspectives of family and friend care partners are also valuable. In a review of non-wearable technologies Narasimhan et al. found that most study participants did not report any issues with acceptability for home based monitoring [12]. In contrast, Schütz et al. found that although older adult study participants did not express privacy concerns with the use of sensors, they did express concern about the appearance of the sensor devices and described desire to retain control over their sensor data first before allowing access to a family care partner [24]. Therefore, it is recommended that future research examining the implementation of Toch Sleepsense consciously gather and highlight the LTCF residents own perspectives as well as the resident and family experiences of the impact of the devices on quality of life and quality of care in the LTCF.

Future research should move beyond feasibility to assess the clinical effectiveness and sustainability of sensor-based technologies in long-term care. This includes measuring resident-level outcomes such as falls and sleep quality, conducting longitudinal studies on sustained use and workflow impact, and exploring differences across facility types and infrastructure levels. Further investigation should also include non-clinical staff perspectives and examine how organizational readiness, training models, and interprofessional collaboration influence successful implementation.

## 5. Conclusions

This study explored the early experiences and impacts of implementing Toch Sleepsense across three LTCFs in Western Canada, showcasing the technology’s potential to improve care in LTCFs. Further, this study highlights the critical organizational factors required for successful use of non-wearable bed sensor technologies. Staff consistently acknowledged Toch Sleepsense as valuable for enhancing resident safety through real-time monitoring of falls, sleep patterns, and vital signs, which substantially reduced the frequency of intrusive nighttime checks and facilitated more personalized, proactive care planning. In well-supported settings, these technological benefits translated clearly into improved workflow efficiency, staff confidence, and reduced emotional burden, which was particularly noticeable during night shifts, when monitoring responsibilities typically increase staff stress and workload.

However, these benefits were contingent upon stable network infrastructure, comprehensive staff training, and active leadership engagement. Connectivity issues, insufficient education on sensor placement and troubleshooting, and unclear implementation roles led to frustration and undermined trust in the system’s reliability. The experiences shared by participants emphasized that technology alone does not guarantee improved outcomes; rather, the organizational context, especially leadership involvement and systematic preparation, is pivotal.

Long-term care organizations considering similar sensor-based monitoring technologies must prioritize robust, ongoing training and reliable technical infrastructure. Equally essential is a leadership-driven approach that actively involves all team members, including clinical, maintenance, and housekeeping staff, to ensure comprehensive integration into daily care routines. Only by addressing these critical organizational elements can the potential of sensor-based technologies be fully realized to support safer, more responsive, and person-centered long-term care environments.

## Figures and Tables

**Table 1:** Sociodemographic Characteristics of Study Participants (*N* = 34).

Characteristic	Category	Online Survey Participants (*N* = 25)	Focus Group Participants (*N* = 9) ^1^
Age	18–34	5 (20%)	2 (33%)
	35–54	16 (64%)	3 (50%)
	55+	4 (16%)	1 (17%)
Gender	Women	23 (92%)	6 (86%)
	Men	2 (8%)	1 (14%)
Job role	Management	-	4 (57%)
	Licensed Practical Nurse	3 (12%)	-
	Care Aide/Healthcare Assistant	17 (68%)	1 (14%)
	Registered Nurse	5 (20%)	2 (29%)
Place of employment	Site A	2 (8%)	2 (29%)
	Site B	4 (16%)	1 (14%)
	Site C	19 (76%)	4 (57%)
Experience at job role	Less than 1 year	4 (16%)	1 (14%)
	1–5 years	10 (40%)	4 (57%)
	5+ years	11 (44%)	2 (29%)
Experience in LTCF	Less than 1 year	3 (12%)	1 (14%)
	1–5 years	6 (24%)	2 (29%)
	5+ years	16 (64%)	4 (57%)
Shift schedules	Day shift	4 (16%)	5 (71%)
	Day shift or Night shift	4 (16%)	1 (14%)
	Night shift	17 (68%)	1 (14%)

^1^ Note: Two focus group participants did not disclose demographic characteristics; therefore, percentages are calculated out of *N* = 7.

## Data Availability

Data are available upon request from the corresponding author.

## Supplementary Materials

The following supporting information can be downloaded at: https://www.mdpi.com/article/10.3390/s25216795/s1: Text S1: Toch Sleepsense evaluation survey administered to staff, Text S2: Semi-structured interview guide used for qualitative interviews with staff participants.

## Abbreviations

The following abbreviations are used in this manuscript:

LTC	Long-Term Care
LTCF	Long-Term Care Facility
