# Supplementary material for: Enhancing Quality of Resident Care and Staff Efficiency Through Implementation of Sensors in the Long-Term Care Setting: A Multi-Site Mixed-Methods Study
Source: Sensors (Basel). 2025 Nov 6;25(21):6795. doi: 10.3390/s25216795 (PMC12609713; doi:10.3390/s25216795)
Supplement: Supplementary file 1 [file sensors-25-06795-s001.zip › S1.pdf]

Northern Health

Certificate of Ethical Approval for Harmonized Minimal Risk Behavioural Study

Also reviewed and approved by:

- **University of Northern British Columbia**

**Principal Investigator:**	**Primary Appointment:**	**Board of Record REB Number:**	**UBC REB Number:**
Shannon Freeman	UBC/UBCO - Faculty of Health and Social Development/UBCO - Nursing	**Board of Record:** Northern Health	H23-04033

Study Title:
Implementation, Evaluation, and Expansion of the Toch Sleepsense Technology

Study Approved: June 18, 2024 **Expiry Date: June 18, 2025**

Research Team Members: N/A

Sponsoring Agencies: N/A

Documents included in this approval:

Document Name	Version	Date
**Protocol:**		
TochTech Sleepsense Study Research Proposal	1.0	April 18, 2024
**Consent Forms:**		
Staff Consent Form and Questionnaire	2.0	May 13, 2024
Staff Focus Group Consent Form	2.0	May 13, 2024
Management Focus Group Consent Form	2.0	May 13, 2024
**Advertisements:**		
Questionnaire Staff Poster	1.0	April 18, 2024
Focus Group Staff Poster	1.0	April 18, 2024
Focus Group Management Poster	1.0	April 18, 2024
**Questionnaire, Questionnaire Cover Letter, Tests:**		
Staff Focus Group Script	1.0	April 18, 2024
Management Focus Group Script	1.0	April 18, 2024
Staff Consent Form and Questionnaire	2.0	May 13, 2024
Demographic Questionnaire	1.0	April 18, 2024
Gift Card Questionnaire	1.0	April 18, 2024
**Letter of Initial Contact:**		
Staff Questionnaire Invitation	1.0	April 18, 2024
Staff Focus Group Invitation	1.0	April 18, 2024
Management Focus Group Invitation	1.0	April 18, 2024
**Other Documents:**		
TochTech Sleepsense Protocol Flowchart	2.0	May 13, 2024
CTAAN Website Info	1.0	April 18, 2024
Confidentiality Agreement	1.0	April 18, 2024
**Other:**		
www.ctaan.ca		

This ethics approval applies to research ethics issues only and does not include provision for any administrative approvals required from individual institutions before research activities can commence.

The Board of Record (as noted above) has reviewed and approved this study in accordance with the

most recent requirements of the Tri-Council Policy Statement: Ethical Conduct for Research Involving Humans (TCPS2).<br><br>The "Board of Record" is the Research Ethics Board delegated by the participating REBs involved in a harmonized study to facilitate the ethics review and approval process.
The application for ethical review and the document(s) listed above have been reviewed and the procedures were found to be acceptable on ethical grounds for research involving human subjects.
**This study has been approved either by the Board of Record's full REB or by an authorized delegated reviewer.**

REBC
