# Supplementary material for: Enhancing Quality of Resident Care and Staff Efficiency Through Implementation of Sensors in the Long-Term Care Setting: A Multi-Site Mixed-Methods Study
Source: Sensors (Basel). 2025 Nov 6;25(21):6795. doi: 10.3390/s25216795 (PMC12609713; doi:10.3390/s25216795)
Supplement: Supplementary file 1 [file sensors-25-06795-s001.zip › S2/TochSleepsense_Consent_FocusGroups_LTCStaff.pdf]

Implementation, Evaluation, and Expansion of the Toch Sleepsense Technology

Draft Information Letter & Consent Form (Long-Term Care Home Staff)

Who is running this study?

Principal Investigator
Dr. Shannon Freeman
Associate Professor, School of Nursing
University of Northern British Columbia
Office phone: 250-960-5154
Email: shannon.freeman@unbc.ca

Study Team
Matt Sargent
Research Manager, School of Nursing
University of Northern British Columbia
Email: matt.sargent@unbc.ca

Study Team
Emma Rossnagel
Research Manager, School of Nursing
University of Northern British Columbia
Email: emma.rossnagel@unbc.ca

Study Team
Aaron Miller
Executive Director (Interim) Seniors Specialized Care Transformation
Interior Health
Email: aaron.miller@interiorhealth.ca

Do I have to agree to be in this study?

Taking part in this study is voluntary; you do not have to take part in this research. You can withdraw from this study at any time. You are also free to not answer any questions that you don't want to. For Interior Health employees, if the study occurs during your work hours, it is your responsibility to get a manager's approval to take part in this research.

If I agree to be in the study, what would happen?

You will participate in a focus group over Zoom, during which you will be asked questions encouraging you to share your thoughts on a range of issues related to the implementation of Toch Sleepsense. The discussion will include, but may not be limited to, the following issues: impact of Toch Sleepsense on your workload, impact of Toch Sleepsense on quality of patient care, accuracy of Toch Sleepsense reports, and perceived strengths and challenges to the implementation of the Toch Sleepsense technology.

If you agree to participate in the focus group by completing the consent form at the end of this document and returning it to the research team, the research team will be in contact with you in the following days and/or weeks about setting up a time for a focus group. Prior to participating in the focus group, you may be asked to answer questions about yourself such as your age, gender, role, and experience in your current role. Answering these questions is completely voluntary. When the focus group is scheduled, you will be emailed a Zoom link for the focus group. The focus group session itself will take about 60-90 minutes.

During the focus group, a member from the research team will ask the group questions about your experiences with Toch Sleepsense. This part of the focus group will be audio-recorded - by clicking "OK" to the meeting being recorded, you are reconfirming that you consent to take part in the research. Some or all of the

participants in the focus group may be known to you. The focus group audio will then be transcribed (written out) afterwards.

Should you choose, you will be able to leave the focus group at any time, without reason.

You may be contacted via email after the focus group (up to 6 months) by the research team if we need to check further whether information presented in reports and/or other writings aligns with what was said in the focus group.

Please note that focus group participation will occur on a first-come first-serve basis: staff who are the first to contact the research team will have first priority to participate in the focus groups. Completing this consent form does not necessarily mean you will be able to participate in the focus group. In the event that the focus group has reached capacity because other staff have completed and returned the form and made arrangements with the research team earlier, you may be unable to participate.

Why should I take part in this study?

Should you participate in the focus group, the views, opinions, and experiences you share with us may help inform policy regarding the future implementation of bed monitoring systems in care homes. Further, your feedback will help us guide and shape technologies for supporting aging adults and care partners. We hope to improve available technologies and supports by getting feedback from the people who are interested in or may use these technologies.

Is there any way that being in the study can be harmful for you?

We do not think there is anything in this study that could be harmful for you. However, please let one of the study staff know if you have any concerns. If, at any point in the study, you feel uncomfortable or upset and wish to end your participation, please let the study staff know. If you would like, we can chat with you after the focus group in a private break-out room on Zoom. We have also provided a list of support resources and phone numbers for you in this document. You will be able to leave the focus group at any time.

Will being in this study help you in any way?

Taking part in this study may not immediately or tangibly help you. However, from what we learn, some of the study findings and subsequent decisions on technology design and implementation may benefit you and others in the future.

Commercialization

The findings from this study may contribute to the development or improvement of TochTech products, which may result in commercial profit for the TochTech Technologies company. Study participants and research team members will not share in this potential commercial profit.

How will your identity be protected?

We will do everything we can to protect your confidentiality. We will encourage everyone attending to not discuss the content of the focus group to people outside the group; however, we cannot control what they may do with the information discussed. It is likely you may know other people who choose to join the conversation, so your anonymity is not guaranteed.

All focus group discussions are conducted on the Zoom platform and hosted by an account managed by the University of Northern British Columbia (UNBC) Enterprise licenses for Zoom. While privacy cannot be

guaranteed for information that is transmitted over the internet, all reasonable efforts to ensure your information security are being taken, including password-protected meeting invites, utilizing the waiting room function, and ensuring that the audio recordings of the focus groups are only stored on password-protected servers at UNBC and accessed only by research team members.

All study related data will be stored on a password-protected server at UNBC. Only members of the research team will have access to the de-identified study data. Data will be retained for a minimum of five years after we have completed all research-related activities.

You can withdraw consent at any time during the study, even if the focus group has already been started. If you do withdraw during the focus group, your data will be confidentially and permanently deleted. However, if you withdraw consent after the focus group discussion has been transcribed (written out) and analysis has begun, it may not be possible to remove all your information.

Will you be paid for taking part in this research study?

If you participate in a focus group, you will a receive a $20.00 e-gift card of your choice (Starbucks, Tim Hortons, or Amazon) in appreciation for taking part in all focus group activities (including the focus group and pre-focus group demographic questionnaire, if applicable), and in recognition of the internet/phone resources required to take part in the focus group.

How will the study results be shared?

The study findings may also be published in academic journal articles, shared through conference presentations, community/public talks, infographics, reports, and on the research team website. Findings may be used as part of student project and thesis work. The findings from this study may contribute to the development or improvement of TochTech technologies; any commercial profit gained will not be shared with participants or research team members.

You may also choose to receive a summary of the study results by providing your contact information on the consent form.

Questions, Concerns or Complaints about the project

If you have any questions about this study, please contact the research manager at Matt.Sargent@unbc.ca or the principal investigator at Shannon.Freeman@unbc.ca.

If you have any concerns or complaints about your rights as a research participant and/or your experiences while participating in this study, contact the UNBC Office of Research at 250 960 6735 or by e-mail at reb@unbc.ca.

Staff Perspectives on Toch Sleepsense Technology in Interior Health Care Homes.

Draft Consent Form

Please read the following carefully:

I have read or been described the information presented in the information letter about the project.

☐YES ☐NO

I have had the opportunity to ask questions about my involvement in this project and to receive additional details I requested.

☐YES ☐NO

I understand that if I agree to participate in this project, I may withdraw from the project at any time up until the report completion, with no consequences of any kind.

☐YES ☐NO

I have been given a copy of this form.

☐YES ☐NO

I agree to be recorded.

☐YES ☐NO

I have experience with Toch Sleepsense at my place of work.

☐YES ☐NO

I have read the consent information and I understand what my participation in the study involves.
Your signature indicates that you consent to participate in this study.

____

Participant’s Signature Date

____

Participant’s Name (print)

____

Research Team Member’s Signature Date

____

Research Team Member’s Name (print)

- Please let us know what type of e-gift card you would like and which email address you would prefer we send it to:

E-gift card type: ☐ Starbucks ☐ Tim Hortons ☐ Amazon ☐ No e-gift card

Please provide the email address you would prefer we send the e-gift card to:

____

- Would you like to receive a brief summary of the study findings?

☐YES ☐NO

Please provide the email address you would prefer we send the summary to:

____

- May we contact you in the future about other aspects of this or new studies?

☐ No, I would not like to be contacted about future studies

☐ Yes, I would like to be contacted about future studies – if yes, please provide an email address

____
