# Supplementary material for: Enhancing Quality of Resident Care and Staff Efficiency Through Implementation of Sensors in the Long-Term Care Setting: A Multi-Site Mixed-Methods Study
Source: Sensors (Basel). 2025 Nov 6;25(21):6795. doi: 10.3390/s25216795 (PMC12609713; doi:10.3390/s25216795)
Supplement: Supplementary file 1 [file sensors-25-06795-s001.zip › S2/TochSleepsense_Consent_Survey_LTCStaff.pdf]

UNBC UNIVERSITY OF NORTHERN BRITISH COLUMBIA

AGE-WELL CTAAN NATIONAL INNOVATION HUB

Implementation, Evaluation, and Expansion of the Toch Sleepsense Technology

Draft Information Letter, Consent Form, & Questionnaire (Long-Term Care Home Staff)

Who is running this study?

Principal Investigator
Dr. Shannon Freeman
Associate Professor, School of Nursing
University of Northern British Columbia
Office phone: 250-960-5154
Email: shannon.freeman@unbc.ca

Study Team
Matt Sargent
Research Manager, School of Nursing
University of Northern British Columbia
Email: matt.sargent@unbc.ca

Study Team
Emma Rossnagel
Research Manager, School of Nursing
University of Northern British Columbia
Email: emma.rossnagel@unbc.ca

Study Team
Aaron Miller
Executive Director (Interim) Seniors Specialized Care Transformation
Interior Health
Email: aaron.miller@interiorhealth.ca

Do I have to agree to be in this study?

Taking part in this study is voluntary; you do not have to take part in this research. You can withdraw from this study at any time. You are also free to not answer any questions that you don't want to. For Interior Health employees, if the study occurs during your work hours, it is your responsibility to get a manager's approval to take part in this research.

If I agree to be in the study, what would happen?

You will complete a questionnaire, in which you will be asked to indicate your level of agreement with several statements covering a range of issues related to the implementation of Toch Sleepsense technology. You will also be asked to provide responses to open-ended questions about your experience with Toch Sleepsense. The question topics will include, but may not be limited to, the following issues: impact on your work efficiency, perceived impact on residents, reliability of the technology, and utility of available information provided by the Toch Sleepsense devices.

Prior to answering the questions related to Toch Sleepsense on the questionnaire, you will be asked to fill in a short demographic survey that will ask you questions about yourself such as your age, gender, and length of time at your current role. Answering these questions is completely voluntary. After this, you will prompted to complete the questionnaire about your experiences with Toch Sleepsense, which will take approximately 10-15 minutes to complete. You will be able to exit the questionnaire at any time, without reason.

Why should I take part in this study?

Should you complete the questionnaires, the views, opinions, and experiences you share with us may help inform policy regarding the future implementation of monitoring systems in care homes. Further, your

feedback will help us guide and shape technologies for supporting aging adults and care partners. We hope to improve available technologies and supports by getting feedback from the people who are interested in or may use these technologies.

Is there any way that being in the study can be harmful for you?

We do not think there is anything in this study that could be harmful for you. However, please let one of the study staff know if you have any concerns. If, at any point in the study, you feel uncomfortable or upset and wish to end your participation, you may exit the questionnaire. We have also provided a list of support resources and phone numbers for you in this document.

Will being in this study help you in any way?

Taking part in this study may not immediately or tangibly help you. However, from what we learn, some of the study findings and subsequent decisions on technology design and implementation may benefit you and others in the future.

Commercialization

The findings from this study may contribute to the development or improvement of TochTech products, which may result in commercial profit for the TochTech Technologies company. Study participants and research team members will not share in this potential commercial profit.

How will your identity be protected?

We will do everything we can to protect your confidentiality. While privacy cannot be guaranteed for information that is transmitted over the internet, all reasonable efforts to ensure your information security are being taken. The questionnaire will be delivered on Survey Monkey, on a UNBC licensed account which has enhanced security features and is hosted on Canadian servers.

All study related data will be stored on a password-protected server at UNBC. Only members of the research team will have access to the de-identified study data. Data will be retained for a minimum of five years after we have completed all research-related activities.

You can withdraw consent at any time during the study, even if the questionnaire has already been started. If you do withdraw consent during the questionnaire, your data will be confidentially and permanently deleted. However, if you withdraw consent after the questionnaire has been completed and analysis has begun, it may not be possible to remove all your information.

Will you be paid for taking part in this research study?

If you participate in this study and complete the questionnaire, you will receive a $10.00 e-gift card of your choice (Starbucks, Tim Hortons, or Amazon) in appreciation for completing the questionnaire, and in recognition of the internet/phone resources required to take part in the questionnaires. We will e-mail the e-gift card to you within one month of the completion of the final questionnaire.

How will the study results be shared?

The study findings may also be published in academic journal articles, shared through conference presentations, community/public talks, infographics, reports, and on the research team website. Findings may be used as part of student project and thesis work. The findings from this study may contribute to the development or improvement of TochTech technologies; any commercial profit gained will not be shared with participants or research team members.

You may also choose to receive a summary of the study results by providing your contact information on the consent form.

Questions, Concerns or Complaints about the project

If you have any questions about this study, please contact the research manager at Matt.Sargent@unbc.ca or the principal investigator at Shannon.Freeman@unbc.ca.

If you have any concerns or complaints about your rights as a research participant and/or your experiences while participating in this study, contact the UNBC Office of Research at 250 960 6735 or by e-mail at reb@unbc.ca.

Staff Perspectives on Toch Sleepsense Technology in Interior Health Care Homes.

Draft Consent Form

Please read the following carefully:

I have read or been described the information presented in the information letter about the project.

☐YES ☐NO

I have had the opportunity to contact the research team to ask questions about my involvement in this project and to receive additional details I requested.

☐YES ☐NO

I understand that if I agree to participate in this project, I may withdraw from the project at any time up until the report completion, with no consequences of any kind.

☐YES ☐NO

I have experience with Toch Sleepsense at my place of work.

☐YES ☐NO

I have read the consent information and I understand what my participation in the study involves.

☐YES ☐NO

- Please let us know what type of e-gift card you would like and which email address you would prefer we send it to:

E-gift card type: ☐ Starbucks ☐ Tim Hortons ☐ Amazon ☐ No e-gift card

Please provide the email address you would prefer we send the e-gift card to:

____

- Would you like to receive a brief summary of the study findings?

☐ YES ☐ NO

Please provide the email address you would prefer we send the summary to:

____

- May we contact you in the future about other aspects of this or new studies?

☐ No, I would not like to be contacted about future studies

☐ Yes, I would like to be contacted about future studies – if yes, please provide an email address

____
